# A Cyclin T1 point mutation that abolishes positive transcription elongation factor (P-TEFb) binding to Hexim1 and HIV tat

**DOI:** 10.1186/1742-4690-11-50

**Published:** 2014-07-01

**Authors:** Nina Verstraete, Alona Kuzmina, Gaelle Diribarne, Van Trung Nguyen, Lydia Kobbi, Monika Ludanyi, Ran Taube, Olivier Bensaude

**Affiliations:** 1 Institut de Biologie de l’Ecole Normale Supérieure, Paris F-75005, France; 2 UMR 8197, Centre National de la Recherche Scientifique, Paris F-75005, France; 3 U1024, Institut National de la Santé et de la Recherche Médicale, Paris F-75005, France; 4 The Shraga Segal Department of Microbiology, Immunology and Genetics, Faculty of Health Sciences, Ben-Gurion University of the Negev, Beer-Sheva 84105, Israel; 5 Present Address CEA, Cadarache 13108, France; 6 Functional Genomics – S2, Institut de Biologie de l’Ecole Normale Supérieure, 46 rue d’Ulm, Paris, Cedex 05 F-75230, France

**Keywords:** CDK inhibition, Genetic mapping of protein-protein interfaces, P-TEFb, Cyclin T, Hexim1, 7SK RNA

## Abstract

**Background:**

The positive transcription elongation factor b (P-TEFb) plays an essential role in activating HIV genome transcription. It is recruited to the HIV LTR promoter through an interaction between the Tat viral protein and its Cyclin T1 subunit. P-TEFb activity is inhibited by direct binding of its subunit Cyclin T (1 or 2) with Hexim (1 or 2), a cellular protein, bound to the 7SK small nuclear RNA. Hexim1 competes with Tat for P-TEFb binding.

**Results:**

Mutations that impair human Cyclin T1/Hexim1 interaction were searched using systematic mutagenesis of these proteins coupled with a yeast two-hybrid screen for loss of protein interaction. Evolutionary conserved Hexim1 residues belonging to an unstructured peptide located N-terminal of the dimerization domain, were found to be critical for P-TEFb binding. Random mutagenesis of the N-terminal region of Cyclin T1 provided identification of single amino-acid mutations that impair Hexim1 binding in human cells. Furthermore, conservation of critical residues supported the existence of a functional Hexim1 homologue in nematodes.

**Conclusions:**

Single Cyclin T1 amino-acid mutations that impair Hexim1 binding are located on a groove between the two cyclin folds and define a surface overlapping the HIV-1 Tat protein binding surface. One residue, Y175, in the centre of this groove was identified as essential for both Hexim1 and Tat binding to P-TEFb as well as for HIV transcription.

## Background

Transcription of most class II genes stops shortly after initiation because RNA polymerase II (RNAPII) is arrested by negative elongation factors (NELF, DSIF). Phosphorylation by the positive transcription elongation factor (P-TEFb) is required to counteract their effect and resume a productive transcription elongation [1]. In particular, recruitment of P-TEFb to the early transcription elongation complex is a critical step for productive HIV genome expression [2]. This recruitment relies on P-TEFb association with the Tat viral protein bound to the TAR domain of the nascent transcript and results in phosphorylation of the Negative Elongation Factors, NELF and DSIF, that prevent elongation of transcription. P-TEFb next phosphorylates the RNAPII Carboxy-Terminal Domain (CTD) and might thus contribute to recruit splicing, cleavage and polyadenylation factors for co-transcriptional pre-mRNA processing [3]. Core P-TEFb comprises a kinase, Cdk9, and a Cyclin T (CycT1 or CycT2). A cellular feed-back loop involving the 7SK small nuclear RNA regulates the activity of P-TEFb [4,5]. *In vivo*, 7SK snRNA forms a core complex with the MePCE and Larp7 proteins [6]. This core 7SK snRNP complex binds the Hexim1 protein and the Hexim1/7SK snRNP in turn contacts CycT1 and inactivates P-TEFb [7-10]. Hexim1 competes with Tat for P-TEFb binding and represses Tat transactivation of HIV transcription [11]. The equilibrium between active and inactive cellular P-TEFb complexes is highly regulated. Following transcriptional arrest, 7SK RNA is trapped by heterogeneous nuclear ribonucleoproteins (hnRNPs), resulting in release of Hexim1 and increased P-TEFb activity [12-14]. Such release might be assisted by interactions with Brd4 [15-17], JMJD6 [18] and Tat [19,20]. Partial 3-D structures of P-TEFb [21,22], Hexim1 [23] and 7SK RNA [24,25] have been established by X-ray crystallography or NMR. Nevertheless, little is known on how these components assemble together. To tackle these questions, an extensive single amino-acid mutagenesis of human Hexim1 and Cyclin T1 residues was performed. Mutants deficient in Hexim1/Cyclin T1 binding were screened by yeast two-hybrid. Combined with 3-D structures obtained by NMR or X-ray crystallography, such strategy determines surface residues involved in protein/protein interactions [26].

## Results

### Randomly generated Cyclin T1 mutations screened by reverse two-hybrid

The ability of Hexim1 proteins to directly bind Cyclin T1 was assayed in a yeast two-hybrid assay in cells where the *ura3* gene is placed under the control of GAL4 regulatory sequences [7]. Cells expressing wild-type Cyclin T1 and wild-type Hexim1 fused to the GAL4 DNA-binding and activation domains respectively grew in a selective medium lacking uracil (LTU), thereby demonstrating a contact between both proteins. 7SK RNA is not required for Cyclin T1/Hexim1 interaction in this test [8]. Consistently, the “ILAA” Hexim1 mutant that is deficient in 7SK RNA binding interacts with Cyclin T1.

**Figure 1:**
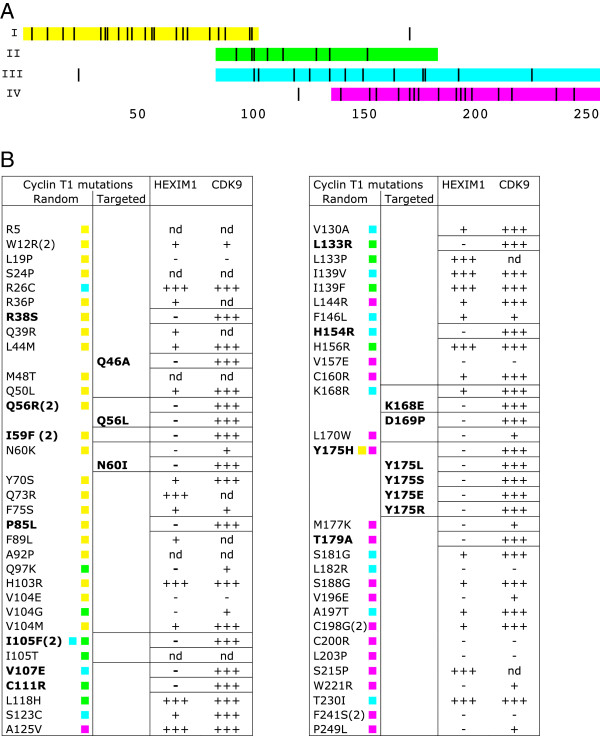
**Cyclin T1 point mutations screened for Hexim1 binding deficiency by yeast two-hybrid. (A)** Distribution of Cyclin T1 single mutations generated by random mutagenesis. Colours mark the sequences that were amplified in four different error-prone PCR reactions: I – from residue 1 to 106 (yellow), II – from 88 to 188 (green), III – from 88 to 261 (cyan) and IV – from 140 to 261 (magenta). The position of single amino-acid mutations obtained following each PCR are highlighted in black. Several mutations are found outside the PCR amplified sequence. **(B)** List of Cyclin T1 point mutations obtained by “Random” or “Targeted” mutagenesis. Numbers in brackets indicate the mutations that occurred more than once. Wild-type interaction (+++), decreased interaction (+) and lack of interaction (−) are deduced from regular, slow or no growth on LTU selective medium, respectively. (nd) mutations have not been validated. Mutations leading to complete loss of Hexim1 binding (−) without affecting Cdk9 binding (+++) are shown in bold. Colours indicate the error-prone PCR reactions providing each mutation.

To identify Cyclin T1 residues that are required for Hexim1 binding, we used random mutagenesis by error-prone PCR followed by a reverse two-hybrid screen in yeast [26,27]. The 5-step procedure was restricted to the 260 N-terminal amino-acids of Cyclin T1 corresponding to the Cyclin Box Domain (CBD) since a previous study had indicated that it comprised the Hexim1 binding sequences [7]. A mutant Cyclin T1 library was generated by PCR amplification of the CBD using error-prone conditions to introduce randomly dispersed mutations (step 1a). In parallel, the CBD sequence was excised from the Gal4BD-CycT1 plasmid by restriction (step 1b). Yeast cells containing the *ura3* gene under the control of Gal4 promoter were co-transformed with Gal4AD-Hexim1, excised Gal4BD-CycT1 plasmids and the error-prone PCR library to allow homologous recombination of Gal4BD-CycT1 in yeast (step 2). 5-FOA is toxic to yeast when the *ura3*-encoded enzyme is expressed and was used as a counter-selective drug. Thus, Cyclin T1 mutations or truncations that prevented Hexim1 binding to Cyclin T1 allowed transformed cells to survive in the presence of 5-FOA. These colonies were selected (step 3) and DNA fragments of the Cyclin T1 CBD were amplified by PCR. Their size was checked by electrophoresis (step 4) and fragments with appropriate size were sequenced (step 5). Performing random PCR mutagenesis on the entire Cyclin box (909 bp) often generated multiple mutations in the same sequence. As multiple mutations complicate the interpretation, smaller Cyclin T1 fragments were amplified by error-prone PCR to increase the yield of single amino-acid mutations. Four fragments using distinct sets of primers were amplified by error-prone PCR and screened independently (Figure 1A). Fragments I, II, III and IV respectively corresponded to Cyclin T1 residues 1 to 106, 88 to 188, 88 to 261 and 140 to 261.DNA fragments from 203 colonies were sequenced (step 5). Among them, 74 had multiple amino-acid changes, premature stop codons or frame-shifts, 38 had no detectable amino-acid change and 68 contained a unique amino-acid mutation. These corresponded to 61 distinct mutations listed as “Random” of 56 residues scattered along the entire Cyclin Box Domain (260 amino-acids) (Figure 1A and B). 9 of those residues were found mutated more than once in the screen suggesting that a significant proportion of the possible point mutations affecting Hexim1 binding had been generated.

### Cyclin T1 mutations involved in Hexim1 binding

Several mutations were found outside the sequence that had been amplified by error-prone PCR (Figure 1A). For instance, the Y175H mutation was obtained once following error-prone amplification of fragment “I” which spans only over residues 1 to 104. Furthermore, many of the 203 sequenced DNA fragments showed no mutation. These observations called for a validation step. The PCR fragments were cloned back into the starting Gal4BD-CycT1 plasmid. A forward two-hybrid assay (growth on selective medium lacking uracil) was then used to validate the loss of Hexim1 binding. Indeed, 30 mutations allowed growth like the wild-type Cyclin T1 (+++), or just showed growth impairment (+) and were not further investigated (Figure 1B). However, 26 out of the initial 61 Cyclin T1 mutants did not grow at all (−) thereby confirming a loss in Hexim1 binding capacity.As P-TEFb is a heterodimer composed of Cyclin T1 and Cdk9, we looked for Cyclin T1 mutations that would not disrupt the Cyclin T1/Cdk9 interaction. Thus, the 26 Cyclin T1 mutants deficient for Hexim1 binding were further submitted to a forward yeast two-hybrid assay for binding to Cdk9. 15 Cyclin T1 mutants deficient (−) for Hexim binding were either completely (−) or partially (+) impaired for Cdk9 binding as well (Figure 1B). However, the reverse two-hybrid experiment still provided 10 Cyclin T1 mutants that had lost detectable interaction with Hexim1 and kept full Cdk9 binding capacity (Hexim (−), Cdk9 (+++), shown in bold in Figure 1B) and were kept for further studies. 9 additional such mutants were generated by targeted mutagenesis as discussed below.

### Mutations leading to Hexim1 binding deficiency cluster along a groove between the cyclin folds

**Figure 2:**
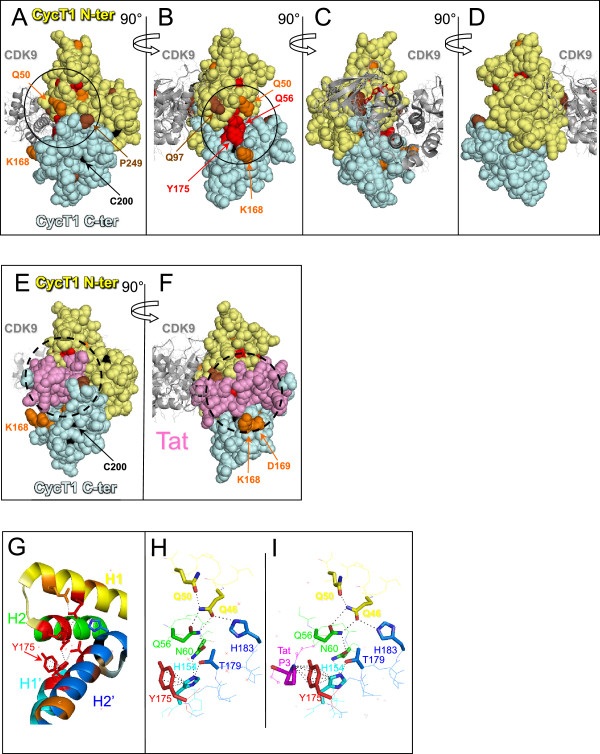
**Positioning of Cyclin T1 mutations on the 3D structure of P-TEFb. (A), (B), (C)** and **(D)** Cyclin T1 mutations were localised on P-TEFb.ATP (pdb3blq) and **(E)** and **(F)** on Tat.P-TEFb.ATP (pdb3MIA) 3D structures. The N-terminal cyclin fold is in pale yellow, the C-terminal one is in pale cyan and Tat is in pink. Mutations are coloured according to their 2-hybrid interaction phenotype (Figure 1B); in red [Cdk9(+++); Hexim1(−)], orange [Cdk9(+++); Hexim1(+)], brown [Cdk9(+); Hexim1(−)] and black [Cdk9(−); Hexim1(−)]. Targeted mutagenesis of K168 and D169 provided [Cdk9(+++); Hexim1(−)] interaction phenotypes. **(G)** Aromatic residues in the funnel formed by helices in N-terminal and C-terminal Cyclin folds. Position of Cyclin T1 mutations on helices H1 (yellow), H2 (green), H1′ (cyan) and H2′ (marine) of P-TEFb.ATP (pdb3blq) structure; **(H)** and **(I)** H-bond network linking the cyclin fold helices to Y175 in P-TEFb.ATP (pdb3blq) and Tat.P-TEFb.ATP (pdb3MIA), respectively.

Despite high divergence in amino-acid sequences, Cyclins share a common conserved cyclin box structure [28]. Cyclin boxes derive from an ancient duplication that generated two cyclin folds. When positioned on the Cyclin T1 3-D structure derived from crystals of P-TEFb [21,22], many residues essential for Hexim1 binding appeared clustered on the Cyclin T1 surface along the groove between the cyclin folds (Figure 2A-D). Mutations of most these residues kept a normal interaction with Cdk9 (orange and red colours) but some showed a partial Cdk9 binding deficiency (brown colour). Cyclin T1 residues such as C200, leading to complete Cdk9 binding deficiency were coloured in black and hardly seen on the surface as they were buried inside the cyclin folds. Furthermore, although Cdk9 interacts mainly with residues in the N-terminal fold (pale yellow) rather than the C-terminal fold (pale blue) of Cyclin T1, several mutations leading to Cdk9 binding deficiency corresponded to residues in the C-terminal Cyclin fold. Mutation of these residues likely lead to major twists in the cyclin box structure.

Importantly, several residues that came out in the screen cluster around tyrosine Y175 that is largely exposed to the solvent and positioned between the two cyclin folds (Figure 2B). Targeted mutagenesis of K168 and D169 residues which are in close proximity to Y175, provided two additional mutants of Cyclin T1 that were impaired for their Hexim1 binding, while efficiently bound Cdk9 (Figure 1B). Noteworthy, the HIV-1 Tat protein binds human Cyclin T1 in the same groove between the cyclin folds that is centred around Y175 as shown on the 3-D structure [22] (Figure 2E and F).

### Conserved binding residues in the *C. elegans* Cyclin T1 and Hexim1 homologues

**Figure 3:**
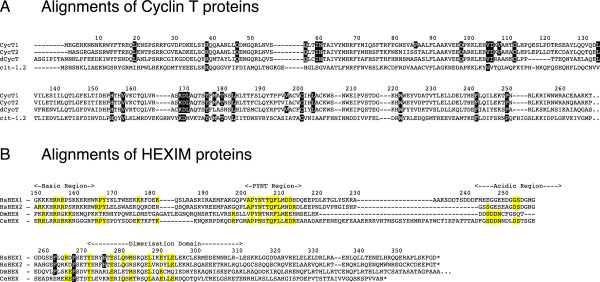
**Conservation of Hexim and Cyclin T proteins. (A)** Alignment of Cyclin T amino-acid sequences from *Homo sapiens* (CycT1 and CycT2); *Drosophila melanogaster* (dCycT); *Caenorhabditis elegans* (cit-1.2)*.* CycT1 residues generated by random mutagenesis that resulted in Hexim1 binding deficiency are highlighted in black. Numbers above the sequences correspond to the a.a. number in the human Hexim1 and Cyclin T1 sequences respectively. **(B)** Alignment of Hexim amino-acid sequences from *Homo sapiens* (HsHEX1 and HsHEX2); *Drosophila melanogaster* (DmHEX); *Caenorhabditis elegans* (CeHEX)*.* Nematode residues conserved in metazoa are highlighted in yellow. Residues highlighted in black are required for Cyclin T1 binding in a two-hybrid assay.

**Figure 4:**
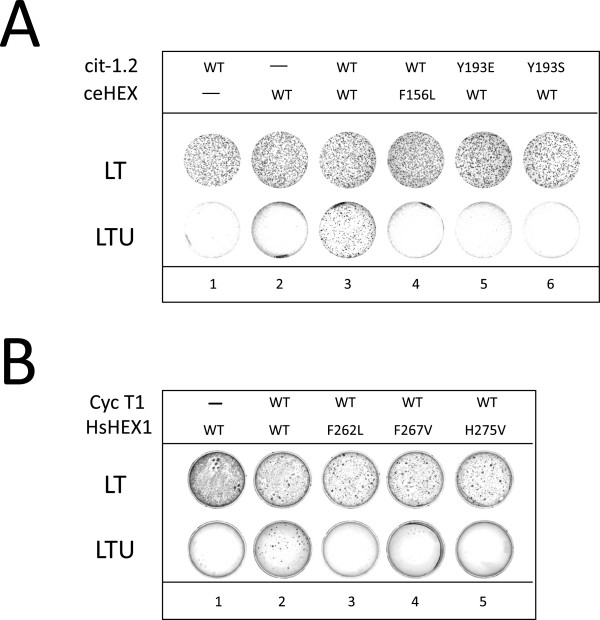
**Two-hybrid assay of nematode Hexim interactions with Cyclin T. (A)** Yeast cells transformed with plasmids expressing wild-type or mutant *C.elegans* Hexim (ceHexim) and Cyclin T (cit-1.2) fused to the Gal4 DNA-binding and Gal4 activation domains, respectively, grew in LT medium. *Ura3* expression induced by interaction between partners is required for growth in LTU medium (lacking uracil). **(B)** Like in A but using human Hexim1 and Cyclin T1 fusion proteins.

Regulation of P-TEFb by Hexim1/7SK RNA has been discovered in human cells and recently described in Drosophila cells [29]. It has been conserved throughout evolution at least from mammals to insects. Apart from one (P85L in human Cyclin T1), mutations disrupting Hexim1 binding without affecting Cdk9 binding correspond to residues conserved in human Cyclin T1 and Cyclin T2 as well as in Drosophila Cyclin T (Figure 3A, highlighted residues). One Cdk9 and two Cyclin T1 homologues (cit-1.1 and cit-1.2) have been characterized in *C. elegans*[30] while a bioinformatic study suggested that Hexim and 7SK RNA homologues are present in nematodes [31]. The putative ceHexim shows very weak sequence homologies with its metazoan counterparts (Figure 3B). Nevertheless, we found that ceHexim and cit-1.2 interact in the two-hybrid assay (Figure 4A, lane 3). Mutations of Y175 abolished human Cyclin T1/Hexim1 interaction in a two-hybrid assay (Figure 1B). Both Y193E and Y193S mutations of cit-1.2 (corresponding to mutations of Y175 in human Cyclin T1) abolished interaction with ceHexim (Figure 4A, lanes 5 and 6).The weak homologies between human and nematode Hexim proteins concern residues in three small known important sequences: the basic region involved in 7SK RNA binding, the PYNT motif and the N-terminus of the dimerization domain already known to be involved in Cyclin T binding. A systematic targeted mutagenesis of conserved residues in the PYNT region and the dimerization domain of human Hexim1 pointed to three new residues that impaired Cyclin T1 binding in a two hybrid assay: F262, F267 and H275 (Figure 4B, lane 3–5). The F156L mutation of ceHexim (corresponding to F267 mutation in human Hexim) also abolished ceHexim interaction with cit-1.2 in the two-hybrid assay (Figure 4A, lane 4), thus paralleling with observations in the human system. These results highlighted the importance of the conserved phenylalanine F267 in Hexim1 and tyrosine Y175 in Cyclin T1 (human nomenclatures). Moreover, they further support the idea that despite their limited sequence homology, nematodes have a true Hexim homologue, interacting with a Cyclin T1 homologue.

### Mutations of Y175 impair Cyclin T1 binding to Hexim1 in human cells

**Figure 5:**
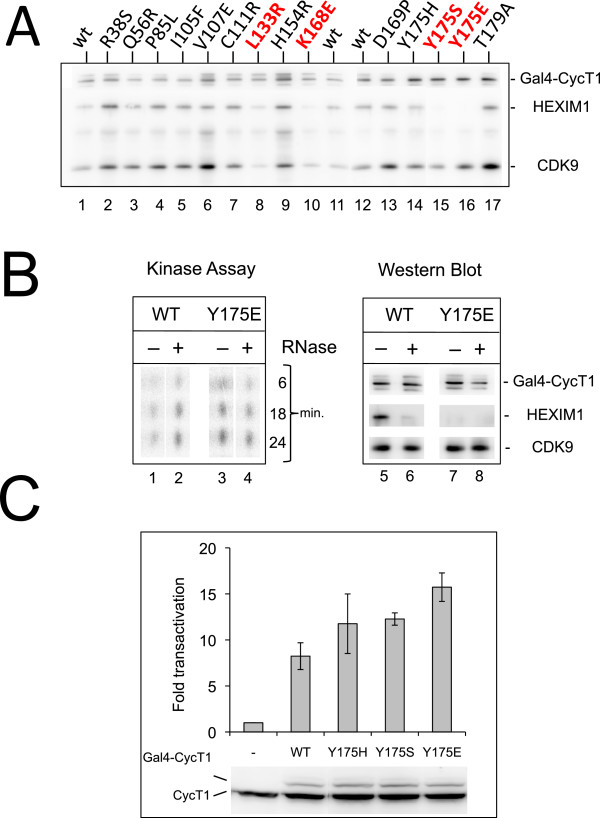
**Functional characterization of Cyclin T1 mutant proteins expressed in human cells. (A)** Coimmunoprecipitation of Cdk9 and Hexim1 with Gal4-CycT1 proteins. Mutants in red are reproducibly deficient for Hexim1 binding. Gal4-CycT1 mutant proteins were transiently expressed in HEK293 cells. Immunoprecipitation was performed with anti-Gal4 antibodies. Immunoprecipitated proteins were detected on Western blot with Cdk9, Cyclin T1 and Hexim1 antibodies. **(B)** CTD kinase assay associated with immunoprecipitated Gal4-CycT1 proteins. (Left) Incorporation of ^32^PO_4_ in a CTD4 peptide increases with incubation time (min.). (Right) Western blots showing that immunoprecipitates used for kinase assays contained the same quantities of Cdk9 and Gal4-CycT1 proteins. **(C)** In vivo assays of P**-**TEFb activity. Expression of a luciferase reporter gene driven by 5 Gal4 UAS upstream a minimal HIV promoter is activated by cotransfected Gal4-CycT1 proteins in HEK 293T cells. Fold transactivations are relative to luciferase activity in cells that do not express Gal4-Cyclin T1 proteins. Data result from three independent experiments with standard error bars. Western blots show proteins detected in lysates of typical experiments using an anti Cyclin T1 antibody. Gal4-Cyclin T1 migrates above the endogenous Cyclin T1.

Mutant Gal4BD-CycT1 proteins that had successfully passed the validating two-hybrid assays [binding to Hexim1 negative (−) and Cdk9 positive (+++)] were next expressed in mammalian cells. Wild-type Gal4-CycT1 expressed in 293 cells co-immunoprecipitated CDK9 and Hexim1 (Figure 5A, lanes 1, 11 and 12) like other previously reported fusion Cyclin T1 [32]. However, most of the 14 Hexim1 (−) Cdk9 (+++) mutations tested did not significantly alter Hexim1 binding. Only 4 Cyclin T1 mutants (L133R, K168E, Y175E and Y175S) were reproducibly impaired in Hexim1 binding relative to Gal4-CycT1 wild-type (lanes 8, 10, 15, 16). The L133R mutant was reproducibly deficient in Cdk9 binding. Perhaps because L133 is buried within the N-terminal cyclin fold, close to the Cdk9 interaction surface. Replacement of leucine by a charged residue might affect the folding of the whole domain. In contrast, K168 and Y175 are exposed to the solvent. K168E, Y175H, Y175S and Y175E mutants associated Cdk9 as efficiently as the wild-type protein. The Y175E mutation exhibited the sharpest deficiency in Hexim1 binding.

To better characterize the Y175E mutation, a CTD-kinase assay was performed on immunoprecipitates. The activity of P-TEFb with wild-type Cyclin T1 was enhanced when RNAse was added to digest 7SK and disrupt Hexim1 interaction [4,5] (Figure 5B, lanes 1,2). In contrast, the kinase activity of P-TEFb with CyclinT1-Y175E was not affected by RNAse (lanes 3,4) and was similar to that of wild type Cyclin T1 after treatment with RNAse.

As an assay for Cyclin T1 mutant function in live cells, we used an assay that would rely on P-TEFb recruitment. A luciferase gene transcription was driven by a minimal HIV promoter, comprising 5 Gal4 binding sites upstream the TATA box. P-TEFb is recruited to and activates transcription from such promoter when Cyclin T1 is fused to the Gal4 DNA binding domain [33,34]. Indeed, wild type Gal4 Cyclin T1 enhanced luciferase production (Figure 5C). Gal4 Cyclin T1s with a mutated Y175 were reproducibly close to two fold more efficient. This was consistent with their higher CTD kinase activity and likely due to an escape from repression by Hexim1.

To conclude, we have generated point mutations in Cyclin T1 that strongly suppress Hexim1 binding capacity and brought stronger P-TEFb activity than wild-type Cyclin T1.

### Mutations of Y175 strongly impair Cyclin T1 binding to HIV Tat

**Figure 6:**
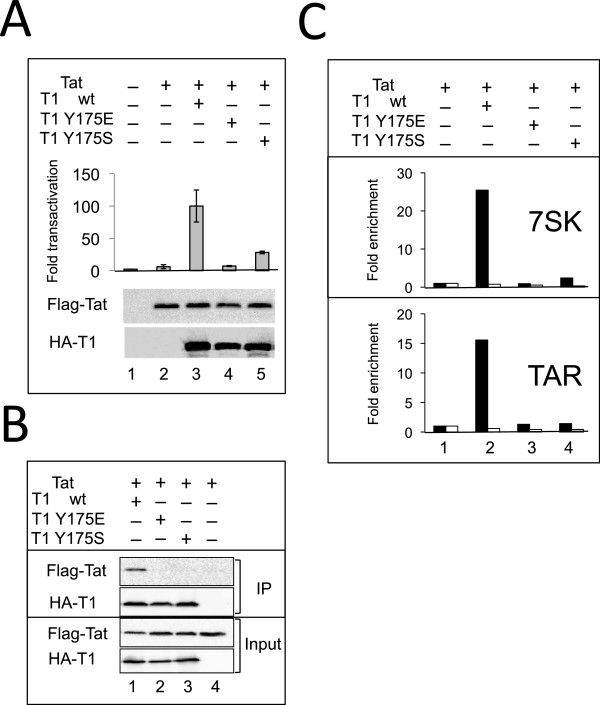
**Y175 Mutations in Cyclin T1 impair binding to 7SK snRNA, HIV TAR and Tat. (A)** CycT1-Y175 mutations abolish Tat transactivation in murine cells - 3T3 murine cells were co-transfected with the Myc-Tat, HA-CycT1 (wild type or Y175 mutant human Cyclin T1 proteins), HIV-LTR-*Photinus* luciferase and a *Renilla* luciferase reporter. 48 hr after transfection, luciferase activity was monitored. *Photinus* luciferase readings were normalized to *Renilla* expression. Fold transactivation are relative to luciferase activity in the absence of both Tat and human Cyclin T1. Rescue of Tat transactivation by wild-type Cyclin T1 was set to 100. Results are representative of the mean value of triplicate wells; error bars show ± SEM. Lower panel shows expression levels of HA-Cyclin T1 in the experiment. **(B)** Impaired association of Cyclin T1-Y175 mutants with HIV Tat. 293T cells were co-transfected with HA-Tagged CycT1-wild type or Y175 mutated, Flag-Tat and GFP expressing plasmids to control transfection efficiency. HA-Cyclin T1 proteins were precipitated with α-HA antibody, separated on SDS-PAGE and analyzed by Western blot with antibodies against their epitope Tags. **(C)** Impaired association of Cyclin T1-Y175 mutants with TAR and 7SK snRNA. 293T cells were transfected with the HIV-LTR-BFP provirus (generating TAR RNA and Tat) and with the indicated HA-Cyclin T1 wild type or mutants. 48 h post transfection RNAs were coimmunoprecipitated with α-HA (■) or non-immune (□) rabbit antibodies and analyzed by RTqPCR using primers specific to either 7SK snRNA or HIV TAR. The signal obtained from RNAs immunoprecipitated in the absence of co-transfected HA-Cyclin T1 was set to 1.

We next investigated the ability of Cyclin T1 mutants to support Tat dependent transcription from the HIV LTR. As murine Cyclin T1 cannot support Tat transactivation, we monitored the rescue of Tat transactivation in 3T3 cells by human HA-Cyclin T1 proteins [35]. Indeed, wild type HA-Cyclin T1 efficiently rescued Tat transactivation in murine cells (Figure 6A). In contrast, both Y175E and Y175S mutant proteins were deficient and did not rescue Tat transactivation. The available 3D structure of P-TEFb reveals that tyrosine Y175 is in the middle of the Tat interacting surface on Cyclin T1 [21,22] (Figure 2, compare 2B and 2F). Therefore, we investigated the capacity of HA-Cyclin T1 proteins to associate Flag-Tat in human cells. Indeed, Flag-Tat associated with the wild type HA-Cyclin T1 (Figure 6B). Whereas neither Y175E nor Y175S mutant HA-Cyclin T1 did. Binding of CycT1 to Tat enhances its affinity to TAR in an *in vitro* assay [36]. Consistently, we found that while Cyclin T1- wild type efficiently bound to HIV TAR, the Y175E and Y175S mutants did not (Figure 6C). Furthermore, Tat also binds 7SK snRNA [37]. Similarly, while CycT1-wild type associated with 7SK snRNA, the Y175E and Y175S mutants did not.

Taken together these data indicate that tyrosine Y175 is critical for both Hexim1 and Tat binding. As a result, the mutant proteins cannot associate with TAR and support Tat-dependent activation of an HIV LTR reporter plasmid.

## Discussion

We have found several point mutations in the Cyclin T1 CBD that impaired P-TEFb/Hexim1/7SK snRNP complex assembly. A two-hybrid screen following random and targeted mutagenesis generated several single amino-acid changes in Cyclin T1 that impaired its binding to Hexim1 (Figure 1A and B). However, out of the 15 mutants impaired for Hexim1 binding in the yeast 2-hybrid, only 4 (L133P, K168E, Y175E and Y175S) were found deficient when expressed in human cells. Some important post-translational modifications might be missing in yeast cells or alternatively, isolated Cyclin T1 in the 2-hybrid system might be more sensitive to mutations than Cyclin T1 bound to its partner Cdk9 when expressed in human cells. Furthermore, binding of Hexim1 to Cdk9 might stabilize its interaction with Cyclin T1. A Cyclin T1 mutant resulting from four substitutions and a deletion, CycT1-U7, had been previously described as unable to bind either Cdk9 or Hexim1 [38]. The Y175 mutations, the Y175E one in particular, showed the sharpest Hexim1 binding deficiency in human cells. It is the first single mutant Cyclin T1 protein reported with strong Hexim1 binding deficiency that still retains normal capacity to bind Cdk9 and displays P-TEFb activity *in vivo*.

### An unstructured Hexim1 peptide located N-terminal to alpha-1 helix is essential for Cyclin T1 binding

We found that replacement of the aromatic F262, F267 and H275 Hexim1 residues by aliphatic apolar amino-acids severely impairs human Hexim1 binding to P-TEFb in a two hybrid assay (this work) or immunoprecipitation from human cell lysates (data not shown). The structural importance of phenylalanine F267 is strengthened by its conservation throughout evolution (Figure 3B). A mutation of the corresponding residue in the very distant *C. elegans* Hexim protein homologue also prevented Hexim/Cyclin T interaction. Human Hexim1 F262 and F267 residues belong to an unstructured peptide according to predictions and NMR data [23]. Of particular interest, p21^Cip1^ and p27^Kip1^ cell-cycle CDK inhibitors [39] as well as the HIV-1 Tat protein, a P-TEFb activator, are also largely unstructured before binding their target [39-41]. The critical involvement of an unfolded domain that appears to be similarly involved in Cyclin T1 recognition by Hexim1, might emerge as a common feature in Cyclin dependent kinase regulation.

### Hexim1 likely binds Cyclin T1 in the groove between the two Cyclin folds

Although they were generated randomly, Cyclin T1 amino-acid mutations leading to Hexim1 binding deficiency in the two-hybrid assay were clearly not distributed at random when positioned on the 3-D structure of human Cdk9/Cyclin T1 (Figure 2A-D). The Cyclin Box Domain of Cyclin T1 comprises two canonical “cyclin folds” each consisting of five helices [21]. Although Cyclin T1 contacts Cdk9 through residues in the N-terminal cyclin fold, many residues leading to both Hexim1 and Cdk9 binding deficiency were found in the C-terminal cyclin fold (Figure 1B). However, none of the residues leading to complete Cdk9 binding impairment was exposed to the solvent, suggesting that these mutations might as well have a negative impact on the overall folding of the Cyclin T1 helices and that the conformation of the C-terminal Cyclin fold impacts the conformation of the N-terminal cyclin fold.Several (Hexim1 (−), Cdk9 (+++)) mutated residues were found on the surface, exposed to the solvent. They were clustered around the groove between the two cyclin folds (Figure 2A-D), likely defining a contact interface between Hexim1 and Cyclin T1. The other residues impairing Hexim1 binding were either (i) in the vicinity of other solvent-exposed residues identified in the screen, suggesting that they could correctly orientate the Hexim1-binding amino-acids at the surface or (ii) positioned and oriented within helical interfaces inside the cyclin box, suggesting that their mutations had major effects on the overall maintenance of the Cyclin T1 stability or dynamics.

### Cyclin T1 Y175 at the tip of a hydrogen-bond network connecting both cyclin folds

Tyrosine Y175 was found to be the most critical residue for Hexim1 binding in human cells. It is located at the tip of hydrogen-bond network linking the two cyclin folds. Noteworthy, it adopts distinct conformations in crystals obtained in the presence of DRB [42] or flavopiridol [21] (Additional file 1: Figure S1). It is close to H154 and T179 that also came out in the screen (Figure 2G and H). The six aromatic carbon atoms forming the aromatic ring of Y175 are located at an average distance of 4 Å from carbon atom 5 of the imidazole ring of histidine H154. Such distance and relative orientations are consistent with a Pi hydrogen bond [43] linking the carbon 5 of H154 to the aromatic ring of Y175 (Figure 2H). Supporting the importance of the H154 to Y175 Pi-bond, a targeted replacement of Y175 by a serine (Y175S) lacking a side-chain aromatic ring also provided a strong Hexim1 binding deficiency phenotype. The 3-D structure further suggests that H154 is itself engaged in a Pi-bond with the side-chain amide hydrogen of asparagine N60 and engages a conventional hydrogen bond with the side-chain oxygen of threonine T179 (Figure 2H). N60 further engages a hydrogen bond with Q56. Q56 also engages hydrogen bonds with Q50 and Q46. Mutations of T179, H154, N60, Q56 and Q50 all came out in the screen. N60, Q56, Q50 or Q46 were next replaced by targeted mutagenesis with residues with aliphatic side-chains. Such replacements suppress H-bonds without introducing charges that might have lead to major conformational changes. The resulting mutant proteins also lost the capacity to interact with Hexim1 and yet retained full capacity to interact with Cdk9. Furthermore, the I59F mutation that introduces a bulky residue next to N60 came out twice in the screen. It might have affected the positioning of N60 thus preventing it to form appropriate hydrogen bonds. This network is important for Hexim1 but not for Cdk9 binding as 7 out of its 8 residues came out in our screen. Thus, tyrosine Y175 is at the tip of a hydrogen bond network involving 8 residues holding together four helices. Helices (H1, H2) belong to the N-terminal whereas helices (H1′, H2′) belong to the C-terminal cyclin fold (Figure 2G). Rigidity of the cyclin box might be important for Hexim1 binding.

### Overlap between Hexim1 and HIV-1 Tat binding interfaces on Cyclin T1

The structure of complexes formed by Cyclin T1 and some of its partners have been solved by X-ray crystallography. The putative Hexim binding surface does not overlap the Cyclin T1 contact surface with the AFF4 subunit of the Super Elongator Complex (SEC) [44,45]. In contrast, the same groove in Cyclin T1 that is between the cyclin folds where Y175 is positioned, is important for binding of P-TEFb to Tat [22] or to Hexim1 (Figure 2E and F). We also identified two residues (K168 and D169) that were impaired for Hexim1 binding, but appear off limits to the Tat and AFF4 binding surfaces. The Q46 and Q50 Cyclin T1 residues that belong to the Y175 H-bond network are also likely involved in Cyclin T1 binding to Tat. Replacing these residues as well as F176 (located next to Y175) by alanines impaired Tat activation [46]. The overlap between Tat and Hexim1 interfaces on Cyclin T1 accounts for their mutually exclusive binding [7,10]. The Tat Proline P3 nitrogen belonging to the peptide bond also forms a Pi-bond with Y175 (Figure 2I). The deficient binding of Tat to both the Y175E and Y175S Cyclin T1 mutants supports an important contribution of this Pi-bond in Cyclin T1 binding to Tat. As a possible consequence, the Cyclin T1 Y175E and Y175S mutant proteins are deficient in supporting HIV LTR transactivation by Tat.

## Conclusion

To summarize, an extensive mutagenesis of both Hexim1 and Cyclin T1 provided insight into how these proteins interact with each other. Our findings illustrate the power of genetics in mapping interfaces in protein-protein interactions [26]. Cyclin T1 mutations impairing Hexim1 binding were found to be clustered on the Cyclin T1 surface overlapping with the HIV-1 Tat protein contact surface [22], thus accounting for Hexim1 and Tat mutual exclusive binding to Cyclin T1. Furthermore, the interaction characteristics of *C.elegans* protein homologues in a two-hybrid assay supported the existence of a functional Hexim homologue in nematodes. Overall, it generated attractive point mutations that retained P-TEFb activity and will be useful to functionally characterize the importance of specific Cyclin T1 interactions in live cells.

## Methods

### Plasmids

The ampicillin resistance gene in pAS∆∆ [47] was replaced by the kanamycin resistance gene by the mini-λ-Red recombination method [48] in NM1100 bacteria (MG1655 mini-λ tet – from Dr. Nadim Madjalani) to provide pASK, a 2 μ yeast vector coding for the TRP1 gene and the GAL4 binding domain. pACT2-Hexim1(181–359) and pACT2-CDK9 were 2 μ yeast plasmids comprising the LEU2 gene and the GAL4 activation domain fused to Hexim1 C-terminal domain or Cdk9 [7]. The Hexim1 cDNA was cloned into pAdRSV-Flag vector as described [7]. An artificial human Cyclin T1 cDNA (CycT1m) was synthesized (Genscript) using the degeneracy of the genetic code introducing many unique restriction sites at convenient positions for genetic engineering. The full-length Cyclin T1m cDNA was cloned between Msc1/BamH1 in pASK to provide pASK-CycT1m comprising the TRP1 gene and the GAL4 DNA binding domain fused to CycT1m. The same sequence was cloned between BspE1/BamH1 in the pEGFP-C1 (ClonTech) expression vector to provide pEGFPC1-CycT1m. The Gal4 DNA binding domain was inserted between AfeI/EcoRI from pEGFPC1-CycT1m to provide pCMVGal4-CycT1m. HA-Tagged wild type or mutant Cyclin T1 Cyclin box (a.a. residues from 1 to 280) were cloned into pHAGE-CMV-IRES-puromycin lentivector between NotI/BamH1 restriction sites to provide pHAGE-HA-CycT1 plasmids. pG5-38-HIV-Luc derives from pG5-38-HIV-CAT [33]. pHIV-LTR-Luc (*Photinus* Luciferase) reporter, pCDNA3-Flag-Tat, pCDNA3-Myc-Tat and the lentiviral proviral vector expressing LTR-Tat-BFP have been described previously [38]. pRL *Renilla* luciferase reporter plasmid was from Promega.

### Yeast two-hybrid assays

MaV103 yeast cells (*MAT***a***SPAL10::URA3 leu2-3,112 trp1-901 his3-D200 ade2-101 gal4*D *gal80*D *can1*^r^* cyh2*^r^* GAL1::HIS3@LYS2 GAL1::lacZ@URA3)* lack the genomic *ura3* gene and have an absolute requirement for uracil in the culture medium [27,47]. However, they have an inserted *ura3* gene copy placed downstream the *Gal4* DNA sequences that is activated when a GAL4 DNA binding domain (GAL4BD) fusion protein binds a GAL4 activation domain (GAL4AD) fusion protein. To perform the Hexim1 vs CycT1 interaction assay, Hexim1 C-terminal domain (Hexim1 181–359) and CycT1 coding sequences were fused to GAL4AD and GAL4BD respectively. A positive interaction permits growth on media lacking uracil. As URA3 converts 5-fluoroorotic acid (5-FOA) into a toxic metabolite [27], a positive interaction results in cell death if 5-FOA is added to the medium.

### Cyclin T1 mutant selection

Fragments of the Cyclin T1 Cyclin Box Domain (CBD) were amplified by 30 cycles of error-prone PCR using Taq polymerase (InVitroGen), 100 ng pASK-CycT1m template, 0.4 mM primers, 7 mM Mg^2+^, 0.25 mM Mn^2+^, 1 mM dCTP and dTTP, 0.2 mM dATP and dGTP in 100 μl final volume. Plasmid pASK-CycT1m (suppressing tryptophan auxotrophy and coding for the entire Cyclin T1 protein) was digested within the CBD sequence by two restriction enzymes and the resulting linearized vector was isolated from an agarose gel. MaV103 strain was transformed first with pACT2-Hexim1(181–359) (suppressing leucine auxotrophy) and grown on SC medium lacking leucine. The resulting cells were further transformed with 1 μg of linearized pASK-CycT1m and 3 μg of purified randomly mutagenized PCR product. The PCR product and the gapped plasmid overlapped over 50 nt on both sides allowing homologous recombination. Transformants were plated on SC medium lacking tryptophane, leucine and uracil (MP biomedical) supplemented with 20 g.l^−1^ uracil and 1.5 g.l^−1^ FOA (stock solution 100 g.l^−1^ in DMSO). FOA resistant colonies were harvested after 10 days of growth at 30°C. The CBD coding sequence was amplified by high-fidelity PCR from isolated colonies lysed in 0.07 M NaOH for 10 minutes at 99°C, using primers flanking the CBD and Phusion polymerase (Finnzyme). The PCR products were sequenced and recloned after EcoRI and AflII restriction into pASK-CycT1m and pCMVGal4-CycT1m.

### Cell culture, transfections, co-immunoprecipitation, kinase and luciferase assays

3T3 or HEK 293 cells were cultured in Dulbecco’s Modified Eagle’s Media supplemented with 10% Fetal Calf Serum. They were either transfected using LyoVec (InvivoGen) or Lipofectamine 2000 (Life Technologies). 48 h after transfection, cells were lysed in 10 mM Hepes pH7.9, 10 mM KCl, 200 mM NaCl, 1.5 mM MgCl_2_, 0.2 mM EDTA, supplemented with 1 mM dithiothreitol, 40 units.ml^−1^ RNasin (Promega), protease inhibitor mixture (P-8340; Sigma), 1 mM phenylmethylsulfonyl fluoride, 0.5% Igepal (SIGMA). Proteins were detected with anti-Cyclin T1 (Santa Cruz sc-8127), anti-Cdk9 (Santa Cruz sc-484), anti-Gal4 (Santa Cruz sc-510) or C4 anti-Hexim1 [7]. Tagged proteins were immunoprecipitated with protein A agarose beads. Western blot were imaged with a LAS 4000 CCD camera system (GE Healthcare). For kinase assays, agarose beads after immunoprecipitation were split in two batches, one was analyzed by Western blot, the other one was incubated at room temperature with (YSPTSPS)_4_ peptide (1 μg per assay) and ATP (100 nM, 0.1 μCi per assay). The reaction was arrested at various times by addition of SDS loading buffer. The phosphorylated peptide was separated by SDS PAGE. The gel was autoradiographed and quantified. For luciferase assays, cells were lysed 48 hr after transfection and both *Photinus* and *Renilla* luciferase activities were determined following the supplier’s recommended procedures (Promega).

### RNA co-immunoprecipitation with mutated Cyclin T1

HEK293T cells were transfected with pHAGE-HA-CycT1wt or mutant, pHIV-LTR-Luc and pCDNA-Myc-Tat. pCMV-GFP was added as a transfection efficiency control (90% cells were green after 48 hrs). 48 hr post transfection cells were lysed in 0.5% NP-40; 20 mM HEPES pH 7.8; 100 mM KCl; 0.2 mM EDTA; protease inhibitor cocktail (Sigma); RNAse inhibitor (New England Biolabs). Clarified cell lysates were incubated overnight either with anti-HA antibody (Abcam - ab-9110) or control anti-human IgG. Protein A-Sepharose beads were pre-blocked with bovine serum albumin and yeast tRNA for 2 hr at 4°C. After immunoprecipitation, input samples and the beads were extracted with Tris-Phenol and chloroform followed by ethanol precipitation. cDNAs were made from RNAs using cDNA high capacity kit (Applied Bio-system). Real time PCR used KAPA SYBER GREEN fast mix and TAR or 7SK specific primers.

## Abbreviations

BFP: Blue fluorescent protein; CBD: Cyclin box domain; FOA: Fluoro-orotic acid; NMR: Nuclear magnetic resonance; P-TEFb: Positive transcription elongation factor; RTqPCR: Reverse transcript quantitative polymerase chain reaction; SDS: Sodium dodecyl sulfate; SEM: Standard error of the mean; UAS: Upstream; Gal4: Activating sequence.

## Competing interest

The authors declare that they have no competing interests.

## Authors’ contribution

NV made most of plasmid constructs, conceived and performed the 2-hybrid screen for Cyclin T1 mutations, the mutant Cyclin T1 immunoprecipitations from human cell extracts, the Gal4-Cyclin T1 in vivo transcription assay and contributed to the writing; AK performed experiments to show that Cyclin T1 mutants, Y175E and Y175S, were deficient in supporting NF-kappa B stimulation and Tat activation of HIV transcription; GD performed the 2-hybrid assay for C.elegans proteins; VTN participated to mutant Cyclin T1 immunoprecipitations and performed kinase assays; LK and ML participated to the 2-hybrid assays; RT conceived and discussed experiments concerning HIV transcription; OB conceived the study and wrote the manuscript. All authors read and approved the final version of the manuscript.

## Supplementary Material

Additional file 1: Figure S1 Alternate positions of Y175. **(A)** Comparison between P.TEFb.ATP (pdb3blq) (grey) and P-TEFb.flavopiridol (pdb3blr) (colour). **(B)** Comparison between P.TEFb.ATP (pdb3blq) (grey) and P-TEFb.DRB (pdb3blr) (colour). Y175(red) in pdb3blq rotates by 90° in pdb3blr and pdb3MY1 (pink). Click here for file
