# Supplementary material for: A Cyclin T1 point mutation that abolishes positive transcription elongation factor (P-TEFb) binding to Hexim1 and HIV tat
Source: Retrovirology. 2014 Jul 1;11:50. doi: 10.1186/1742-4690-11-50 (PMC4227133; doi:10.1186/1742-4690-11-50)

Verstraete - Supplemental Figure

Alternate positions of Y175. (A) Comparison between P.TEFb.ATP (pdb3blq) (grey) and P-TEFb.flavopiridol (pdb3blr) (colour). (B) Comparison between P.TEFb.ATP (pdb3blq) (grey) and P-TEFb.DRB (pdb3blr) (colour). Y175 (red) in pdb3blq rotates by 90° in pdb3blr and pdb3MY1 (pink).

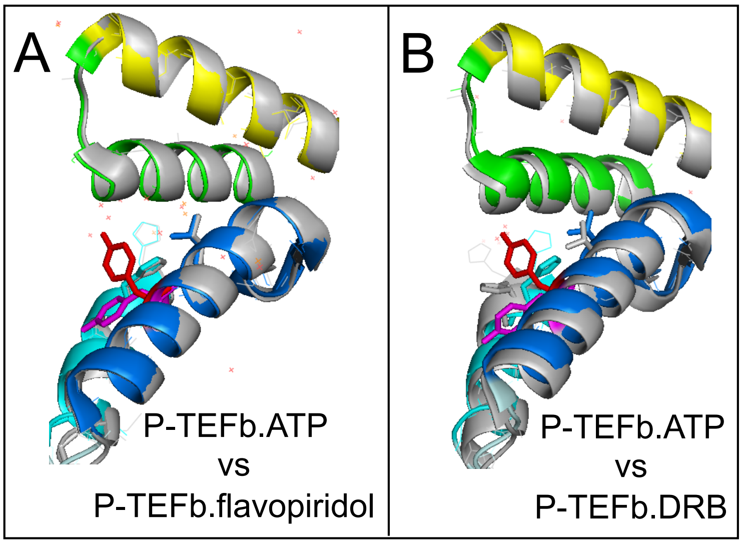

Supplement: Additional file 1: Figure S1 — Alternate positions of Y175. (A) Comparison between P.TEFb.ATP (pdb3blq) (grey) and P-TEFb.flavopiridol (pdb3blr) (colour). (B) Comparison between P.TEFb.ATP (pdb3blq) (grey) and P-TEFb.DRB (pdb3blr) (colour). Y175(red) in pdb3blq rotates by 90° in pdb3blr and pdb3MY1 (pink). [file 1742-4690-11-50-S1.pdf]
